# Antioxidant, anticancer, and anti‐inflammatory potential of Moringa seed and Moringa seed oil: A comprehensive approach

**DOI:** 10.1002/fsn3.4312

**Published:** 2024-07-09

**Authors:** Muhammad Shahbaz, Hammad Naeem, Maryam Batool, Muhammad Imran, Muzzamal Hussain, Ahmed Mujtaba, Suliman A. Alsagaby, Waleed Al Abdulmonem, Ahmed H. El‐Ghorab, Mohammed M. Ghoneim, Mohamed E. Shaker, Mohamed A. Abdelgawad, Entessar Al Jbawi

**Affiliations:** ^1^ Department of Food Science and Technology Muhammad Nawaz Shareef University of Agriculture Multan Multan Pakistan; ^2^ Food Technology Section, Post‐Harvest Research Centre Ayub Agricultural Research Institute Faisalabad Pakistan; ^3^ Department of Food Science and Technology University of Narowal Narowal Pakistan; ^4^ Department of Food Sciences Government College University Faisalabad Faisalabad Pakistan; ^5^ Department of Food Science and Technology, Faculty of Engineering and Technology Hamdard University Islamabad Campus Islamabad Pakistan; ^6^ Department of Medical Laboratory Sciences, College of Applied Medical Sciences Majmaah University AL‐Majmaah Saudi Arabia; ^7^ Department of Pathology, College of Medicine Qassim University Buraidah Saudi Arabia; ^8^ Department of Chemistry, College of Science Jouf University Sakaka Saudi Arabia; ^9^ Department of Pharmacy Practice, College of Pharmacy AlMaarefa University Ad Diriyah Saudi Arabia; ^10^ Pharmacognosy and Medicinal Plants Department, Faculty of Pharmacy Al‐Azhar University Cairo Egypt; ^11^ Department of Pharmacology, College of Pharmacy Jouf University Sakaka Saudi Arabia; ^12^ Department of Pharmacology & Toxicology, Faculty of Pharmacy Mansoura University Mansoura Egypt; ^13^ Department of Pharmaceutical Chemistry, College of Pharmacy Jouf University Sakaka Saudi Arabia; ^14^ Syrian Arab Republic Ministry of Agriculture and Agrarian Reform Damascus Syria

**Keywords:** anticancer, anti‐inflammatory role, antioxidant, mechanisms, *Moringa oleifera*, moringa seed

## Abstract

*Moringa oleifera,* a widely recognized plant more commonly known as moringa, has obtained significant research interest in recent years due to its prospective physiological advantages, including its claimed ability to counteract carcinogenesis. The moringa plant has been found to possess bioactive components that exhibit promising anticancer activities against different human cancers, such as breast cancer, prostate cancer, pancreatic cancer, etc. The cytotoxic properties of moringa seed extracts on cancerous cells have also been provided in this paper along with other notable health benefits. The extracts derived from moringa seeds inhibit cancer proliferation and promote cancer cell apoptosis through multiple signaling pathways. They also stimulated intracellular reactive oxygen species (ROS) production and subsequently induced caspase‐3 activity. The impact of moringin and avenanthramide 2f on the stimulation of caspases 2, 4, 8, and 9 results in reduction in the proliferation of cancer cells. The results reported by research studies hold significant implications for identifying and targeting specific molecular entities that could serve as potential therapeutic targets in search of effective cancer treatments. Furthermore, the flavonoids in moringa seed can remove mitochondrial reactive oxygen species, protecting beta cells and bringing hyperglycemia under control. *M. oleifera* seed oil can reduce the risk of cardiovascular diseases via reduced malondialdehyde (MDA) formation and modulation of cardiac superoxide dismutase (SOD) and glutathione peroxidase (GPx) activity. This article provides a comprehensive summary of the noteworthy discoveries derived from a rigorous investigation that explored into the impact of moringa seeds on the prevention/reduction of various cancers and other complex diseases.

## Graphical abstract

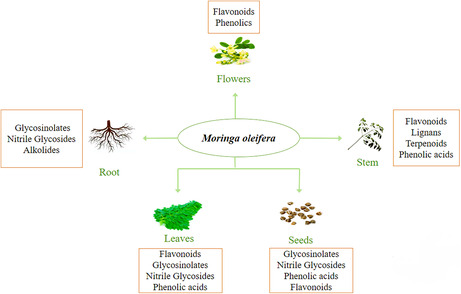

## 1 INTRODUCTION

*Moringa oleifera* is a type of natural medicinal herb that can be used in tropical and subtropical countries because it has a significant potential for treatment of various diseases, such as cancer, diabetes, oxidative stress, and apoptosis. It is an important type of vegetable and it is a member of the Brassicaceae family as well as the Moringaceae family. The moringa plant produces long clusters that resemble drumsticks and are full with many seeds. It is referred to as the “miracle tree” due to the fact that every part of moringa tree is beneficial for human health (Seifu & Teketay, 2020). *M. oleifera* is the most common and widely used plant in the world. Several countries in South East Asia, Africa, Arabia, the Pacific, the Caribbean, and South America are among those that are the native habitat of the plant. Its natural habitat is the sub‐Himalayan highlands that are located in the northwest of India (Olson, 2019). About 20 moringa seeds can be harvested from each fruit or cluster produced by an adult moringa plant. When fully developed, the pods may grow to a height of up to 60 cm and start off green but eventually become brown. The number of seeds that can be found in a single moringa pod can range from 10 to 62 and its weight can be from 25.5 to 37.7 g (Abdel‐Wareth & Lohakare, 2021).

Moringa seeds contain higher quantity of 4‐(alpha‐l‐rhamnopyranosyloxy)‐benzylglucosinolate. The seed of *M. oleifera* has the highest potassium content that is 2357.11 mg/kg, whereas the seed has the lowest calcium content of only 121.14 mg/kg. The seed contains traces of a variety of minerals, including salt, magnesium, and calcium, among others. Threonine, valine, methionine, isoleucine, leucine, phenylalanine, and lysine are the seven essential amino acids that make up *M. oleifera* seed. They contribute 0.824 g/100 g, which is equivalent to 25.65% of the total quantity of amino acids. This amount constitutes 28.8% of the necessary amino acids. *M. oleifera* seeds contain 0.83 g/100 g of seven different hydrophobic amino acids, *M. oleifera* seeds also contain phenylalanine (Liang et al., 2019).

The oil extracted from *M. oleifera* seeds is pale yellow in color and smells very little like hazelnuts. Moringa oil is more resistant to oxidation than other oils, such as olive oil. This property is a result of the many elements that make up moringa oil. *M. oleifera* works on the protection of the epidermis. Because moringa seed oil has some significant ultraviolet (UV)‐protective characteristics, it has been recommended that it be utilized to keep the natural pigmentation of the epidermis intact (Fu et al., 2021). Nevertheless, there are little data to justify its applicability. The antioxidant properties of the seed oil of *M. oleifera* formulations as well as their effects on skin hydration, skin color, and skin viscoelasticity have not been studied. Additionally, there is a lack of information on the appropriate dosage of seed oil of *M. oleifera* to use in formulations (Özcan et al., 2019).

*Moringa oleifera* can be utilized in a variety of contexts. It has economic value since it may be used as a source of nourishment, natural coagulants, natural medicine, animal feed, forestry products, fertilizer, living fence, cultivation, and fuel. Additionally, it can be used to treat wounds naturally. Moringa is considered to be a highly effective medication. This is due to the fact that all of the plant's elements have been utilized to cure a broad variety of diseases and disorders, either on their own or in combination with constituents from other plants (Xu et al., 2021).

**FIGURE 1:**
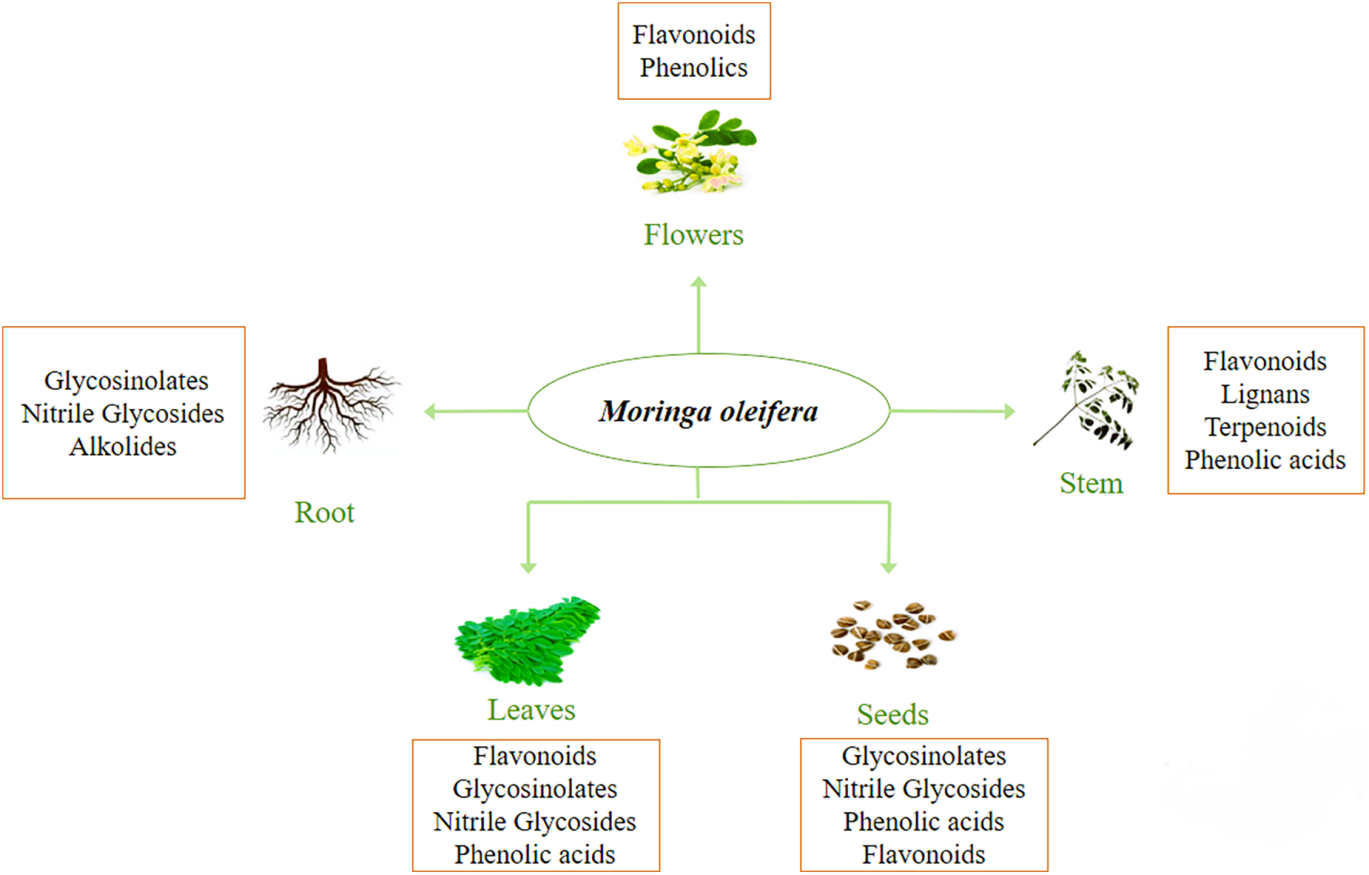
Bioactive compounds in *Moringa oleifera*.

All extracts of moringa exhibit pharmacological qualities and their use is quite extensive. Leaf extracts provide a variety of health benefits, including those that are antiulcerative, hypotensive, and hypocholesterolemic. The effects of the roots were able to improve the analgesic effects of morphine. Antispasmodic, antibacterial, anticancer, and anti‐inflammatory are few of the medicinal properties of seeds. Moringa seeds also contain antioxidant, antidiabetic, and antiobesity properties (Shija et al., 2019). Figure 1 shows the bioactive compounds in different parts of *M. oleifera*.

### 1.1 Moringa seeds

The globular seeds of *M. oleifera* have a diameter of around 1 centimeter (cm). They have three wings that, on average, measure between 0.4 and 0.7 cm in width and have a weight of 0.3 g. Their length ranges between 2 and 2.5 cm. The nucleus accounts for between 70 and 75% of the total weight (Moulin et al., 2019).

The oil makes up around 36.7% of the total weight of the seed. The process of cold‐pressing seeds only allows for the extraction of 69% of the total oil that is present in the seeds. The average concentration of protein in the seed is 31.4%, while the average concentrations of carbohydrates, fiber, and ash are 18.4%, 7.3%, and 6.2%, respectively (Leone et al., 2016).

**FIGURE 2:**
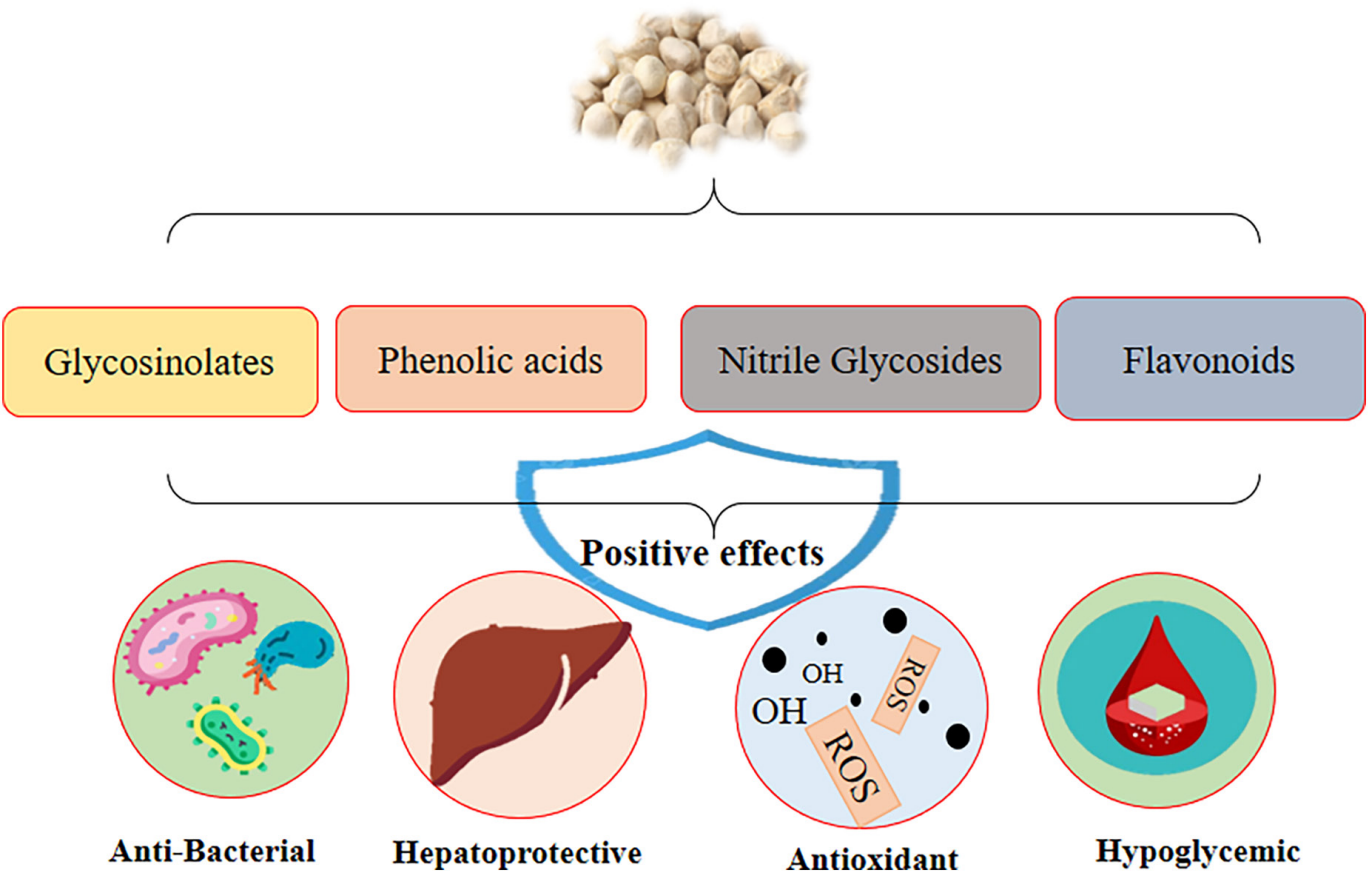
Health benefits of *Moringa oleifera* seeds.

For treatment of ulcers, impaired vision, stomach pain, joint pain, and to aid in digestion, traditional medicine employs raw or crushed *M. oleifera* seeds. Numerous bacterial and fungal species have been discovered to be effectively inhibited by antibacterial properties of the ethanolic seed extract. *M. oleifera* seeds have been discovered to be effective antioxidants, which are able to lessen oxidative damage that is linked to cancer and aging. Numerous bioactive substances have been extracted from *M. oleifera* seeds and it has been revealed that they contain anticancer potential. *M. oleifera* seeds have hepatoprotective, anti‐inflammatory, and antifibrotic effects (Leone et al., 2016). Health benefits of *M. oleifera* seeds are shown in Figure 2.

**FIGURE 3:**
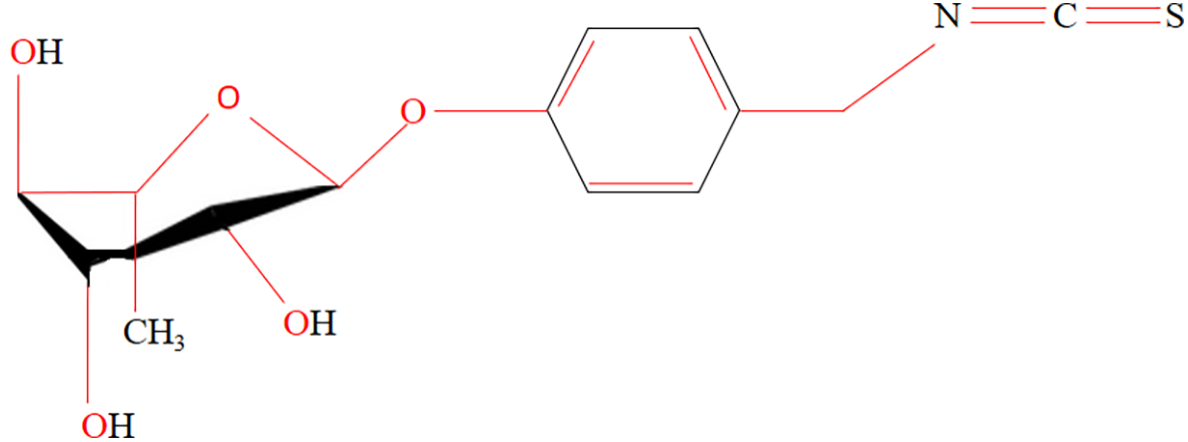
Chemical structure of moringa isothiocyanate‐1 (MIC‐1).

The antioxidant, medicinal, and nutritional properties of the moringa seed extract are due to high quantities of cysteine, methionine, and antioxidants like β‐carotene and vitamins C and E may be responsible for these advantageous benefits, which can be both direct and indirect. Because of its chelation and antioxidant qualities, it also offers defense against oxidative stress caused by hazardous metals. The high phenolic content, ascorbic acid, and isothiocyanate derivatives found in *M. oleifera* seed extract, either directly or indirectly, support the prevention of inflammatory disorders. Significant improvements in both enzymatic and nonenzymatic markers of oxidative stress in the blood and liver point to a dual mechanism in which the seeds directly scavenge free radicals and enhance antioxidants while also indirectly reducing oxidative stress caused by arsenic by lowering the arsenic burden. The ability of *Moringa oleifera* seed extract (MOSE) to modulate biotransformation enzymes and promote detoxification is related to its cancer‐preventive properties. The seed extract exhibits strong hepatoprotective and antibacterial properties. MIC‐1, or 4‐(methylsulfinyl)‐butyl isothiocyanate, is the major bioactive compound found in moringa seeds. It belongs to a class of natural compounds called isothiocyanates, which are known for their various health benefits. The chemical structure of MIC‐1 consists of an isothiocyanate group (−N=C=S) attached to a four‐carbon chain with a methylsulfinyl group. MIC‐1 has been shown to have antibacterial and antifungal properties against various pathogens, e.g., *Escherichia coli*, *Staphylococcus aureus*, etc. MIC‐1 also exhibits antioxidant activity and helps protect cells from damage by free radicals. MIC‐1 may also have anti‐inflammatory properties. Some studies have shown that MIC‐1 may have anticancer properties. It can inhibit the growth and proliferation of cancer cells (Islam et al., 2021). Figure 3 presents the chemical structure of moringa isothiocyanate‐1 (MIC‐1).

#### 1.1.1 Moringa seed oil

It is well known that *M. oleifera* was utilized back in ancient times. It is well known that the ancient Egyptians made use of oil that was produced from fully developed seeds. The potential of the oil to shield skin of Egyptians from the diseases and damage caused by the region's tough environment was highly valued by the residents of the country. After some time, the ancient Greeks and Romans uncovered the oil's medicinal properties and began using it to protect their skin. The oil is perfect for aromatherapy and massage because it does not dry out the skin and does not weigh too much. Because of its exceptional capabilities in the realm of cosmetics, ancient Egyptians made regular use of it as conditioner for both the hair and the body. Ancient civilizations, such as the Egyptians, Greeks, and Romans, all made use of it to extract flower scents for use in perfumery. Floral smells that have been extracted are well preserved after extraction (Athikomkulchai et al., 2020).

It is well documented and accepted that *M. oleifera* seed oil has been shown to reduce inflammation and it exhibits antifungal effects. The oil that is obtained by crushing seeds of *M. oleifera* has traditionally been used to alleviate earaches. Moringa seed oil is added to some soap formulations in Africa to increase lather stability and cleaning effectiveness (Fu et al., 2021).

Seed oils are typically extracted from seeds by methods like cold‐pressing or expeller pressing and solvent extraction. These methods rely on mechanical pressure to squeeze out the oil and solvent extraction is carried out. Seed oils are used in cooking as a frying medium, salad dressing, or flavoring agent. Seed oils are rich in fatty acids (triglycerides), offering nutritional benefits. They have a complex fatty acid profile with varying amounts of saturated, monounsaturated, and polyunsaturated fats. Essential oils are extracted from various plant parts (e.g., leaves, flowers, and roots) using steam distillation, expression, or solvent extraction. Essential oils are highly concentrated in volatile aromatic compounds like esters and phenols. These give essential oils their characteristic strong scent and therapeutic properties, although these claims require further research.

#### 1.1.2 Bioactive components in moringa seed and moringa seed oil

Moringa seeds contain a variety of bioactive compounds including oleic acid, linoleic acid, all essential amino acids, antioxidants such as flavonoids, polyphenols, and good amount of vitamins and minerals. Moringa seed oil has a variety of biomolecules that could be beneficial to health. Moringa seed oil has been shown to contain a variety of bioactive components, including tocopherols, sterols, phenolic acids, flavonoids, and alkaloids. By analyzing the oil with a technique called gas chromatography–mass spectrometry (GC–MS), it is found that moringa seed oil contains significant amounts of oleic acid, linoleic acid, palmitic acid, and large number of phenolic compounds and flavonoids. The antioxidant and anti‐inflammatory characteristics of moringa seed oil are due to the presence of high concentrations of tocopherols, sterols, and phenolic chemicals in the oil (Gharsallah et al., 2021).

The quantity of α‐tocopherol found in moringa seed oil is 15.22 ± 2.99 mg/100 g and this value is obtained by high‐performance liquid chromatography (HPLC) analysis of moringa seed oil. By using gas chromatography (GC), it is determined that the moringa seed oil contains 58.12 mg of brassicasterol, 47.38 mg of campesterol, and 28.94 mg of stigmasterol. The fatty acid found in the highest concentration in moringa seed oil is oleic acid (71.57%). This is followed by the palmitic acid (8.22%), then stearic acid (5.25%), and behenic acid (4.15%) (Athikomkulchai et al., 2020).

Fatty acids, sterols, phenols, and tocopherols make up the bulk of the components that are present in moringa seed oil. Oleic acid, ferrulic acid, β‐sitosterol, and α‐tocopherol are present in the moringa seed oil. Moringa seed oil contains a variety of bioactive components. Moringa seed oil also contains high quantities of antioxidants so it shows extraordinary therapeutic and protective qualities (Gharsallah et al., 2021).

## 2 ANTIOXIDANT ROLE OF MORINGA SEED AND MORINGA SEED OIL

Free radicals are key players in the progression of many chronic illnesses, including cancer, rheumatoid arthritis, cardiovascular disease, and hypertension. Free radicals can harm the primary biological components, such nucleic acids, lipids, proteins, and carbohydrates. Free radicals are the results of both regular aerobic metabolism and metabolic interactions with poisons, medications, and other substances. It has been demonstrated that dietary polyphenols have a significant impact on human health. They are organic antioxidants that are naturally found in plants and provide protection from free radicals when ingested, which is crucial for maintaining good health (Jahan et al., 2018). Moringa seed exhibits antioxidant effects. In a research, Moringa seed flour that has been defatted was studied for its phenolic, antioxidant, and antibacterial properties. According to the findings, the antioxidant and antibacterial activities of the bound phenolic extract were much higher than those of unbound phenolic extract. Furthermore, the extract's extractability of phenolic components was significantly higher as well. The phenolic extracts that were bound and those that were not had IC_50_ (half‐maximal inhibitory concentration) values of 0.9 and 14.9 mg/mL, respectively, when it came to their ability to scavenge DPPH radicals. When compared to free phenolic extract, which had a minimum inhibitory concentration that ranged from 0.117 to 0.191%, the effectiveness of bound phenolic extract ranged from 0.06 to 0.157%. These findings suggest that moringa seeds have the potential to be a rich source of the antioxidant and antibacterial agents in the food as well as for the agricultural and pharmaceutical industries (Carrera‐Chávez et al., 2020).

In another experiment, it was found that seven hydrophobic amino acids and seven essential amino acids (threonine, methionine, isoleucine, valine, leucine, phenylalanine, and lysine) are present in *M. oleifera* seeds and hydrophobic amino acids help the *M. oleifera* seeds to contain antioxidant activity. A pH of 6.7, a temperature of 50°C, and a time duration of 300 min were shown to be the most favorable conditions for the hydrolysis of *M. oleifera* seed protein by flavorzyme. The polypeptide fraction that has a molecular weight of 3.5 kDa is, as a result, the most powerful antioxidant and it has the potential to be further developed into liver‐protective dietary supplements, health goods, functional foods, cosmetics, and medicines (Liang et al., 2019).

In the latest research, it was discovered that aqueous extract of *M. oleifera* seed contained more total phenolic compounds i.e. 90.97 ± 0.134 mg/g of gallic acid equivalent (GAE) than other two extracts of methanol and acetone when three extracts of methanol, acetone, and water of *M. oleifera* seed were screened for the free‐radical scavenging activity and their total phenolic, tannin, and flavonoid contents. Significant antioxidant activity was seen in the water extract, which was confirmed by the type of phytochemical present in the extract. There is evidence to suggest that plant flavonoids have strong antioxidant action. Additionally, it was discovered that the *M. oleifera* seed water extract included more total flavonoids than the other two extracts (221.76 ± 0.221 mg/g (milligrams per gram) of quercetin equivalent (QE)). It was found that this was due to high concentration of the flavonoids and other polyphenolic substances (Jahan et al., 2018).

Moringa seed oil also contains antioxidant properties. In a different investigation, the ferric form of the Fe^3+^/ferricyanide complex was reduced to ferrous form in the presence of the reductants (antioxidants) in the examined samples. The production of Perl's Prussian blue at 700 nm was then measured in order to monitor the Fe^2+^. With increasing concentration, both samples and the *M. oleifera* essential oil gained reducing power. This suggests that *M. oleifera* essential oil may interact with the free radicals, changing them into more stable molecules and stopping the chain process that produces reactive oxygen species. The optimal concentration of *M. oleifera* essential oil that has the highest reducing ability is 1.4 mg/mL. In the following order: Butylated hydroxyanisole (BHAl) > Butylated hydroxytoluene (BHT) > *M. oleifera* essential oil > Trolox > ascorbic acid, reducing power of *M. oleifera* essential oil and BHT reduced. The phenolic content of *M. oleifera* seeds essential oil as well as the presence of thymol, eugenol, and butylated hydroxytoluene may be responsible for its antioxidant properties (Hussein et al., 2014).

In a recent investigation, the capability of *M. oleifera* seed oil to scavenge the free radicals was evaluated using the 2,2‐diphenyl‐1‐picrylhydrazyl (DPPH) technique. Free‐radical molecule‐based reagent called DPPH was used. The decreased DPPH absorption reveals the compound's free‐radical scavenging ability. It has been discovered that *M. oleifera* seed oil helps to reduce DPPH. The quantity of the moringa seed oil and vitamin E improved the free‐radical scavenging activity. Vitamin E and moringa seed oil had inhibitory concentrations of 121.9 mg/mL and 110.4 mg/mL, respectively, that inhibited 50% of free radicals. As positive controls, gallic acid (GA) (0.5 mg/mL) and vitamin E (435 mg/mL) were able to reduce the amount of DPPH free radicals by 93.37 ± 0.15% and 90.61 ± 0.51%, respectively (Athikomkulchai et al., 2020).

In a similar investigation, moringa seed oil's in vitro antioxidant activity was evaluated using a variety of different assays that measured its ability to scavenge free radicals. When compared to control vitamin E (1 mg/mL in chloroform) as well as peanut and tea seed oils (5–35 mg/mL in chloroform), the antioxidant capacity of moringa seed oil was significant. Moringa seed oil also had the lowest peroxide value. It was revealed that the ability of moringa seed oil to scavenge hydroxyl radicals and superoxide anions is more than those of commercial tea oil and peanut oil. Additionally, the capacity of moringa seed oil to scavenge DPPH radicals is better than those of commercial tea oil and peanut oil. It was found that the antioxidant potential of moringa seed oil is higher than those of commercial tea oil and groundnut oil (Chen et al., 2022).

## 3 ANTI‐INFLAMMATORY ROLE OF MORINGA SEED AND MORINGA SEED OIL

Inflammation is a host defensive system that provides protection against pathogens, stressors, and tissue damage. It also plays a significant role in the evolution of many chronic diseases, such as ulcerative colitis, diabetes, atherosclerosis, and arthritis. Nitric oxide (NO), cytokines, nuclear factor kappa B (NF‐κB), and Nuclear factor erythroid 2‐related factor 2 (Nrf2) are two important transcription factors that are involved in the inflammatory response. Research has been done to explore whether or not moringa seeds have any possible anti‐inflammatory qualities. According to the findings of a recent study, the anti‐inflammatory effects of *M. oleifera* seeds are due to a compound called nizazirin. Moringa seeds have the potential to be employed as a dietary supplement for the treatment of inflammatory conditions (Sayed et al., 2022).

In a separate research, the anti‐inflammatory capabilities of *M. oleifera* ethanolic seed extract were studied. It was revealed that the extract can slow down or stop the process of protein denaturation, which is a natural consequence of inflammation. According to the findings of the research, the use of *M. oleifera* seeds in traditional medicine for the treatment of inflammation is supported by the strong anti‐inflammatory characteristics that these seeds possess (Cui et al., 2019).

According to the findings of a research, the seeds of *M. oleifera* can be utilized in the diet as an anti‐inflammatory supplement. Researchers believe that the anti‐inflammatory and antioxidant activities of the seeds are due to the presence of flavonoids, phenolic acids, and glucosinolates in the seeds. It has been suggested that the seeds may be a source of anti‐inflammatory compounds (Ma et al., 2020).

In a study, a chemical known as a glucosinolate containing an isothiocyanate functional group (MIC‐1, 1) was found in high concentrations in the ethanolic seed extract of moringa. This compound has been revealed to have anti‐inflammatory effects. The scientists evaluated its bioaccessibility and bioavailability using a human intestinal model and rodent serum, because there was a lack of metabolic characterization. According to the findings, in contrast to other isothiocyanates, this substance shows minimum change during absorption and possesses appropriate bioavailability and bioaccessibility for a prospective therapeutic treatment (Wolff et al., 2020).

In a recent research, the NCI‐H292, human epidermoid carcinoma‐2 (HEp‐2), and HT‐29 cancer cell lines, as well as murine erythrocytes, were subjected to investigate anti‐inflammatory properties of the aqueous seed extract. In particular, the extracts were able to prevent the formation of NO, tumor necrosis factor alpha (TNF‐α), and interleukin‐1 (IL‐1) in murine macrophages that had been activated with lipopolysaccharide. A mouse model of carrageenan‐induced pleurisy was used and the aqueous seed extract was found to be effective in preventing leukocyte migration. There was also a reduction in the levels of myeloperoxidase, NO, TNF‐α, and IL‐1. According to the authors, the extract brought about a reduction in the quantity of leukocytes that were present in the alveoli (Varadarajan & Balaji, 2022).

Researchers analyzed anti‐inflammatory effects of *M. oleifera* ethanolic seed extract with the use of a murine model of colitis. The researchers found that the extract significantly decreased inflammation in the colon and speculated that the anti‐inflammatory action was due to the isothiocyanates that were present in the extract (Famurewa, Aja, et al., 2019; Famurewa, Asogwa, et al., 2019).

In addition to this, the oil extracted from the moringa seeds also contains anti‐inflammatory qualities. An experiment was conducted to evaluate the anti‐inflammatory effects of the seed oil from the *M. oleifera* plant. The production of pro‐inflammatory cytokines, which are chemicals that are released by cells of the immune system and impact other cells, was significantly reduced as a result of the oil's application. The seed oil of *M. oleifera* may possess anti‐inflammatory capabilities (Edeogu et al., 2020).

According to the findings of another investigation, the seed oil of the *M. oleifera* plant exhibits significant anti‐inflammatory as well as analgesic qualities. The researchers had put forth a theory that the anti‐inflammatory flavonoids and phenolic acids found in the oil were the ones responsible for these advantages. *M. oleifera* seed oil was found to reduce inflammation and increase antioxidant status in a mouse model of colitis. It was revealed that the oil may 1 day be utilized in the treatment of inflammatory bowel illnesses (Famurewa, Aja, et al., 2019; Famurewa, Asogwa, et al., 2019).

A similar research found that the oil extracted from seeds of the *M. oleifera* plant has anti‐inflammatory effects when applied to a mouse model of acute lung damage. Moringa seed oil has potent anti‐inflammatory capabilities, as shown by the oil's capacity to significantly decrease pulmonary inflammation and damage (Shamlan et al., 2021). According to the findings of a similar study, the seed oil from the *M. oleifera* plant decreased inflammation and oxidative stress in a mouse model of arthritis. The researchers guessed that the oil would be beneficial for arthritic patients (Famurewa, Aja, et al., 2019; Famurewa, Asogwa, et al., 2019).

## 4 ANTICANCER ROLE OF MORINGA SEED AND MORINGA SEED OIL

According to data provided by the World Health Organization (WHO), cancer ranks as the second most common cause of death globally, accounting for an estimated 8.8 million mortalities. In the next 20 years, there will likely be a 70% increase in the incidence of cancer. Around 70% of cancer mortalities take place in low‐ and middle‐income nations, most likely due to rising pollution levels, longer life expectancies, a lack of healthcare facilities, and expensive anticancer medications. Creating anticancer medications from organic materials, such as plants, is one strategy to overcome these problems and may result in more cheap medications for low‐ and middle‐income nations (Khor et al., 2018). Moringa contain anticancer qualities. In a research, the chromatogram of GC–MS analyses of moringa extracts (leaves, bark, and seeds) indicated a number of phytoconstituents. The majority of these substances have anticancer activity in cancer cell line models as well as in in vivo testing conditions (Wu et al., 2021). In a recently conducted investigation, it was found that the aqueous extract of *M. oleifera seed* was responsible for initiating the apoptotic pathway in HeLa cells. According to the results of the MTT (3‐(4,5‐dimethylthiazol‐2‐yl)‐2,5‐diphenyl‐2H‐tetrazolium bromide) experiment, mitochondria could have a role in the ability of the extract to begin and promote apoptosis in cancer cells. Isothiocyanates was discovered that a concentration of 70 μg/mL yields an IC_50_ value. The primary bioactive chemicals were also present in the aqueous fraction. These findings demonstrate the anticancer capabilities of this drug and its relative nontoxicity to healthy, normal lymphocytes (Adam et al., 2023).

In another recent experiment, it was discovered that sirtuin‐1 (SIRT1) and B‐cell lymphoma 2 (BCL2), two proteins involved in the apoptotic process, expressed less protein as a result of the pro‐apoptotic action of water extract on *M. oleifera* seeds. The ability of oil–water extract of *M. oleifera* seeds to distinguish between controlling proliferation and death of cancer cells and healthy cells may be attributed to microRNAs (mRNAs) that are found in the extract. It was hypothesized that microRNA in the extract can distinguish between healthy cells and cancer cells and regulate the proliferation of healthy cells and apoptosis of cancer cells (Potestà et al., 2019).

In a similar research, researchers found that the essential oil that was isolated from moringa seeds had a strong inhibitory effect on the growth of numerous cell lines. Depending on the applied oil concentration, all tested cell lines, including HepG2, HeLa, Michigan Cancer Foundation‐7 (MCF‐7), Cancer coli‐2 (CACO‐2), and L929 cells, were considerably affected. Additionally, depending on the type of cell, different cell lines responded differently to the treatment with oil. It was revealed that the impact was depending on concentration and the HeLa and HepG2 cells were the ones that were damaged the most severely. The findings revealed that the essential oil from Moringa seeds may have cytotoxic effects on various cancer cell lines (Elsayed et al., 2016).

Moringa seed oil has been shown to inhibit the growth of tumors. By employing the MTT cell viability assay, it was determined whether or not the extract under investigation was harmful to MCF‐7 (breast carcinoma), HepG2, HCT‐116 (human colorectal carcinoma), HEp‐2, and HeLa (cervical carcinoma) cells. The findings of the investigation show that moringa seed oil displays noteworthy cytotoxic effects. The potent cytotoxic activity of the moringa seed oil is due to the presence of thymol, eugenol, and butylated hydroxytoluene, while the phenolic content of the essential oil extracted from moringa seeds may be responsible for the oil's therapeutic qualities (Hussein et al., 2014).

### 4.1 Colon cancer

Moringa ethanolic seed extracts have anticancer properties, namely in colorectal lines, where they reduce colony formation and cell motility. After treatment with moringa leaf and bark extracts, a decrease in cell viability, an increase in apoptosis, and an enrichment of the G2/M phase were detected. Moringa extracts have the potential to cure aggressive types of colorectal cancer (Zunica et al., 2021). Similarly, moringa seed and seed oil possess anticancer properties against colon cancer and numerous studies have been done to understand better the mechanisms and possible impacts.

In a recently conducted study, the C57BL/6J mouse model of the ulcerative colitis was used to investigate effects of a *M. oleifera* ethanolic seed extract with significant concentration of Moringa isothiocyanate‐1 (MIC‐1) on the Nrf2‐signaling pathway, which is known to moderate anti‐inflammatory signals. The extract contains a higher concentration of MIC‐1. Moringa seed extract exhibited encouraging outcomes in both acute and chronic ulcerative colitis groups. The results suggest that moringa seed extract may be able to prevent the development of ulcerative colitis to colon cancer (Abou‐Hashem et al., 2019).

In a similar study, by using a C57BL/6J mouse model of ulcerative colitis, the effects of *M. oleifera* ethanolic seed extract were studied on the Nrf2‐signaling pathway, which is known to mediate anti‐inflammatory signals. *M. oleifera* seed extract is known to reduce inflammation. Patients with acute and chronic ulcerative colitis responded favorably to moringa seed extract. This suggests that moringa ethanolic seed extract may be effective in avoiding the development of colon cancer as a result of ulcerative colitis (Kim et al., 2022).

In a similar experiment, it was found that moringa seed oil triggers apoptosis in Caco‐2 and HCT‐116 colorectal cancer cells by causing mitochondrial malfunction; however, this process does not have any effect on normal cells (Abd‐Rabou et al., 2016).

### 4.2 Liver cancer

Moringa plays an important role in the prevention of liver cancer. An unusual nutraceutical research was conducted, in which the effects of moringin obtained from moringa seeds on the Hep3B liver cancer cell line, either on its own or in conjunction with avenanthramide 2f generated from oats, were investigated. Myrosinase is the enzyme that is responsible for the breakdown of glucomoringin into moringin. Caspases 2, 4, 8, and 9 are activated when moringin and avenanthramide 2f are present, which results in an inhibition of the proliferation of Hep3B cells. Moringin was able to initiate intrinsic apoptosis pathway by upregulating caspases‐2 and ‐9, increasing the amount of intracellular reactive oxygen species and decreasing the activity of the BIRC5 (baculoviral inhibitor of apoptosis protein (IAP) repeat containing 5) pro‐survival gene (Antonini et al., 2018).

According to a recent study, the methanolic extract of moringa seeds reduced the multiplication of cell lines derived from A549 (lung), 502713 HT‐29 (colon), HEp‐2 (liver), and IMR‐32 (neuroblastoma). The sulforhodamine B (SRB) assay was carried out in order to determine whether or not the moringa methanolic seed extract has antiproliferative qualities. Prior to the application of the SRB stain, each cell line was treated with 100 g/mL of seed extract and kept for a total of 48 hours. The study found that the extract had strong antiproliferative activity against three different cell lines: A549 (80% inhibition), 502713 HT‐19 (95% inhibition), and IMR‐32 (93% inhibition). On the other hand, the proliferation of HEp‐2 cells was not affected in any way by the seed extract. Therefore, the anticancer characteristics of seed extract did not have an effect on all cell lines, but they did have an effect on some (Khor et al., 2018).

Moringa seed oil also plays an important role in the protection of human body from liver cancer. In a research, it was discovered that the active ingredients in *M. oleifera* seed oil can reduce hepatotoxicity and prevent the risk factors for hepatocellular carcinoma (HCC) by acting as an antifibrosis agent through the mechanism of antioxidative reactions. The objective of the research was to determine how changes in liver's shape, histological changes, and circulating levels of damage markers linked to the development of hepatic fibrosis. Additionally, using the molecular docking method, the molecular interaction between a bioactive component of *M. oleifera* seed oil and a liver damage marker was studied. For 8 weeks, carbon tetrachloride (CCl_4_) and corn oil dosed at 1 μL/1 g were intraperitoneally injected into BALB‐c mice aged 6–8 weeks. The antioxidant content of *M. oleifera* seed oil was shown to have the capacity to lessen the progression of HCC brought on by the induction of fibrogenic substances. This mechanism was predicted by factors, such as decreased liver injury, inflammatory response, and fibrosis rate inhibition. In order to stop the progression of HCC, *M. oleifera* seed oil can therefore be suggested as a viable preventative agent (Susanto et al., 2022).

### 4.3 Breast cancer

Moringa seed reduces the proliferation of breast cancer cells. In an experiment, breast cancer cells MDA‐MB‐231 were used to test effectiveness of the seed and leaf extracts of moringa, respectively. It was found that therapeutic potential of the leaf extract was stronger than that of the ethanolic seed extract since apoptosis was found only in the group that had been treated with ethanolic leaf extract, and the cell cycle progression was arrested at the G2/M phase (Al‐Asmari et al., 2015).

In another investigation, MCF‐7 breast cancer cells were treated after grinding *M. oleifera* seeds into a fine powder and then extracting the plant chemicals by using ethanol. It was found that *M. oleifera* ethanolic seed extract had a strong inhibitory effect on the growth of the cell line (Adebayo et al., 2017). In addition, essential oil of the *M. oleifera* seed was shown to have significant cytotoxic action against MCF‐7. Further study and analysis of this essential oil is necessary in order to discover the specific process (Elsayed et al., 2015).

Research conducted both in vivo and in vitro has shown that the oil extracted from moringa seed has anticancer effects. According to the findings of one study, the multiplication of breast cancer cells can be reduced by up to 80% when moringa seed oil is used. Apoptosis, also known as programmed cell death, was induced in cancer cells as a direct result of the oil's presence. In addition to this, it was found that the oil was able to suppress the expression of genes that are related to proliferation and spread of the cancer cells (Elsayed et al., 2015).

In addition, it was found that while providing healthy baby hamster kidney fibroblast cells (BHK‐21) cells with minimal cytotoxicity, *M. oleifera* seed oil nano‐micelles were found to offer a novel therapeutic approach for breast cancer (MCF‐7 cell line) through the use of mitochondrial‐mediated apoptosis (Abd‐Rabou et al., 2016).

### 4.4 Renal cancer

*Moringa oleifera* can prevent and help treat renal cancer. It was found that moringa seeds contain MIC‐1 that has the strongest growth‐inhibitory effects on the 786‐O renal cell carcinoma (RCC) cells among the 30 different types of cancer cells. Furthermore, MIC‐1 (10 μM) was not hazardous to the normal renal (human kidney 2 (HK2)) cells but dramatically reduced the proliferation of five RCC cell lines, including 786‐O, 769‐P, OSRC‐2, SK‐NEP‐1, and human renal adenocarcinoma (ACHN) cells. Additionally, MIC‐1 decreased the expression of matrix metalloproteinase (MMP)‐2 and MMP‐9, as well as the ability of 786‐O and 769‐P cells to migrate and invade. Furthermore, in 786‐O cells and 769‐P cells, MIC‐1 triggered apoptosis, cell cycle arrest, elevated B‐cell lymphoma protein 2 associated X (Bax)/B‐cell lymphoma protein 2 (Bcl‐2) ratio, and decreased the expression of cell cycle‐related proteins. Protein‐tyrosine phosphatase 1B (BPTP1B) activity was decreased by MIC‐1 by connecting to its active site through the hydrogen bonding and hydrophobic interactions, according to investigations of small‐molecule interactions with PTP1B and molecular docking. Additionally, by preventing PTP1B‐mediated activation of Src/Ras/Raf/ERK (non‐receptor protein tyrosine kinase/rat sarcoma/rapidly accelerated fibrosarcoma/extracellular signal‐regulated kinase) signaling pathway, MIC‐1 could decrease proliferation and migration of 786‐O cells. In vivo tests revealed that MIC‐1 significantly enhanced the Bax/Bcl‐2 ratio in tumor tissues and considerably prevented the formation of mouse tumors. Additionally, in Hep‐G2 cells, A431 cells, and HCT‐116 cells, MIC‐1 had no impact on PTP1B‐dependent Src/Ras/Raf/ERK signaling pathway (Xie et al., 2022).

In recent research, it was found that *M. oleifera* seed oil might protect the kidneys of rats from the nephrotoxicity in cancer that is produced by 5‐fluorouracil (5‐FU). On days 8 and 9, the mice were given an intraperitoneal dosage of 5‐FU (75 mg/kg), as well as an oral prophylactic dose of moringa seed oil (5 mL/kg BW/day), to prevent nephrotoxicity. The rats were removed after a period of 48 h. Possible causes of 5‐fluorouracil‐induced renal damage include the overexpression of nuclear factor kappa B (NF‐κB), renal‐inducible nitric oxide synthase, and cytokines, as well as the activation of the apoptotic cascade via the upregulation of caspase‐3. In addition, the activation of the apoptotic cascade may lead to the activation of caspase‐3. On the other hand, the prophylactic treatment of moringa seed oil resulted in a reduction in serum toxicity indicators, a considerable boost in antioxidant defense and antiapoptotic and anti‐inflammatory effects in the kidney. The histological changes served as a corroborating evidence for the biochemical findings. The results of study suggest that oxidative stress, pro‐inflammatory effects, and apoptosis are all factors that contribute to 5‐FU‐induced nephrotoxicity. The ability of moringa seed oil to block the changes is essential for the therapeutic therapy of 5‐FU‐induced nephrotoxicity in cancer patients (Famurewa, Aja, et al., 2019; Famurewa, Asogwa, et al., 2019).

### 4.5 Neuroblastoma

Moringa seeds are used for the treatment of neuroblastma. MIC‐1 that is present in moringa seeds is frequently used for the treatment of neuroblastoma. Researchers discovered that MIC‐1 can stop growth of the malignant cell lines by triggering apoptosis, or programmed cell death, using the SH‐SY5Y human NBL cell line. One of the strongest pro‐survival pathways implicated in the development of NBL and linked to a bad prognosis is phosphatidylinositol 3‐kinase (PI3K), protein kinase B (Akt), and mammalian target of rapamycin (mTOR) pathway (Sui et al., 2014).

In a similar research, SH‐SY5Y neuroblastoma cells were treated with an isothiocyanate called glucomoringin, which was extracted from moringa seeds. The treatment boosted the Nrf2, superoxide dismutase type 1 (SOD‐1), and NADPH‐quinone oxidoreductase 1 (NQO1) pathways. In a dose‐ and time‐dependent way, moringin was able to block the process of cell division. Moringin caused an increase in the expression of p53, p21, Bax, caspase‐3, and caspase‐9, which stopped the cell cycle in the G1 phase (Jaafaru et al., 2019).

In an experiment, CCF‐STTG1 human astrocytoma cells, moringin was shown to activate p53 and Bax and inhibit Bcl‐2 and regulate the important transcription factor Nrf2. Moringin's ability to shield SH‐SY5Y neuroblastoma cells from the damaging effects of oxidative stress gives rise to the possibility that it might be useful in the treatment of neurological illnesses (Rajan et al., 2016).

In recent research, it was found that MIC‐1 complexed with the α‐cyclodextrin can reduce levels of phospho‐phosphatidylinositol 3‐kinase (p‐PI3K), phospho‐mammalian target of rapamycin (p‐mTOR), and phospho‐protein kinase B (p‐Akt) linked to this signaling pathway, inhibiting the signaling pathway and reducing SH‐SY5Y cell viability. In addition, MIC‐1 has the ability to block the PI3K/Akt/mTOR‐activated mitogen‐activated protein kinase (MAPK) pathway, which is essential for regulating a variety of cellular processes, such as cell proliferation, survival, and apoptosis. Additionally, MIC‐1 can boost the expression of p53 and p21 and encourage caspase‐3 cleavage, both of which encourage apoptosis in SH‐SY5Y cells (Giacoppo et al., 2017).

**FIGURE 4:**
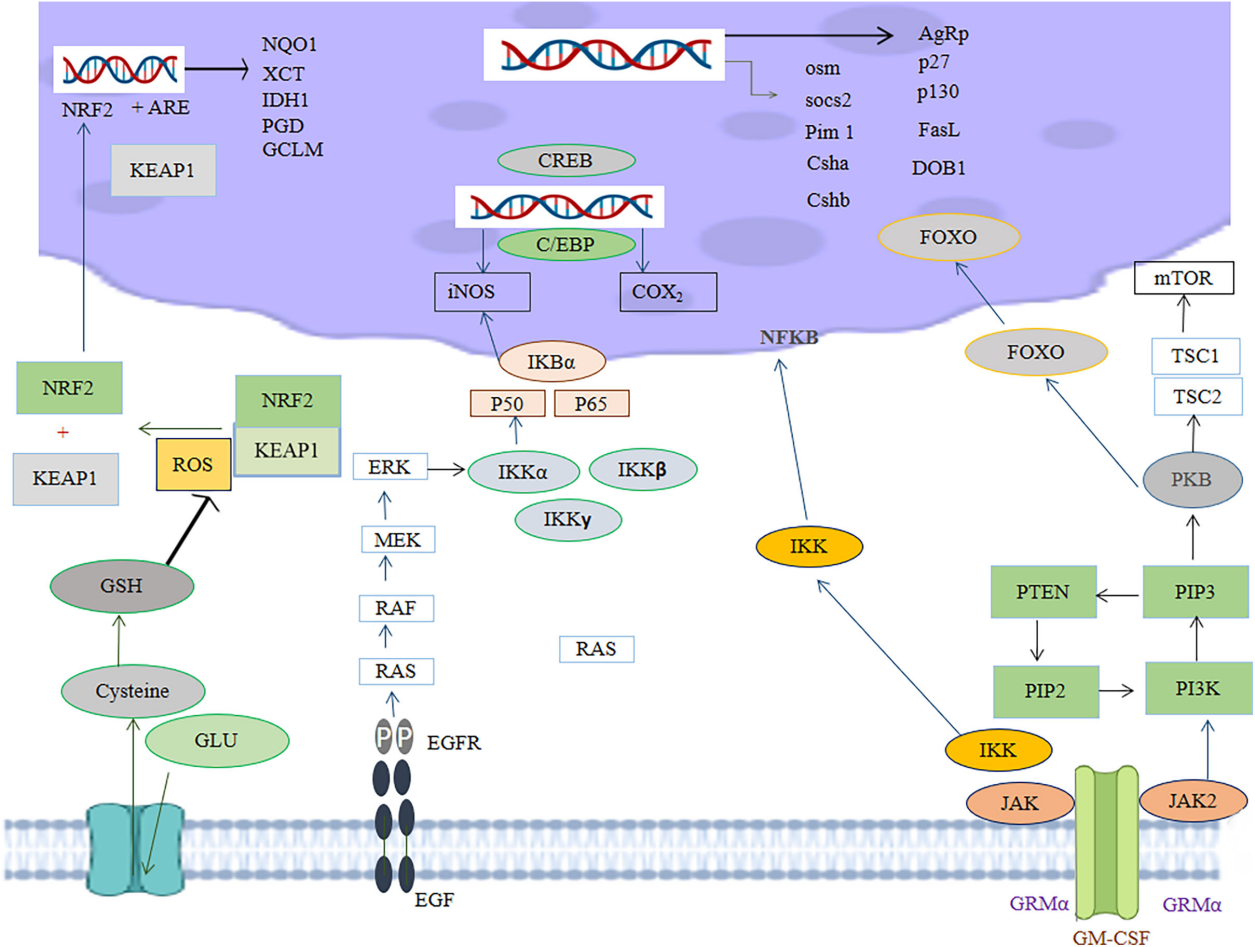
Cellular pathways’ modulatory role of *Moringa oleifera* seed extracts (MOSEs) involved in cancers.

In addition, it was discovered that MIC‐1 can affect how cells normally advance through the cell cycle, increasing the proportion of cells in G2 and S phases while decreasing the proportion of cells in G1 phase and inhibiting NF‐κB's nuclear translocation. Additionally, it was shown that MIC‐1 was able to maintain membrane and the internal structural integrity of differentiated neurons even after hydrogen peroxide had caused oxidative damage, indicating that it has the ability to guard neurons from the oxidative stress‐related neurodegeneration. It is clear from this that further research into targeting NBL is necessary to understand how the drug produces these effects, how it encourages apoptosis, and the precise regulation mechanistic pathways involved in the process (Jaafaru et al., 2018). Figure 4 presents the cellular pathways’ modulatory role of *M. oleifera* methanolic seed extracts involved in cancers. The role of moringa seed and moringa seed oil in the prevention of cancer is shown in Table 1.

**TABLE 1:** Role of moringa seed and moringa seed oil in the prevention of cancer.

Cancer type	Source/intervention	Study type/model	Effect/result	References
Breast	Moringa seed extract	MDA‐MB‐231 cells	Upregulation of SLC2A8 and FoxO6 genes with antiproliferative effect on cancer cells	Zunica et al. (2021)
Breast	Phenolic extract of total moringa	4T1 breast cancer cells	Apoptosis induced by activating caspases‐9 and ‐3 and enhancement in the Bax/Bcl‐2 ratio	Yousefirad et al. (2023)
Colon	Moringa seed extract	Human colorectal carcinoma cells (HCT‐116)	Moringa extracts produced inhibitory action in colon cancer cells (HCT‐116)	Aldakheel et al. (2020)
Colon	Ethanolic extract of moringa	Colorectal cancer (CRC) cell lines T84, SW480, HCT‐15, and HT‐29	Reduction of proliferation in multicellular tumor spheroids of HCT‐15 cells	Mesas et al. (2021)
Liver	Moringa seed extract	Tumor necrosis factor (TNF)‐α and the interleukin (IL)‐1β	Moringa seed extract can reduce expression of the IL‐1β and TNF‐α	Rezkita & Dhelima (2019)
Liver	Moringa fruit extract	HepG2 cells	Reduction in cell viability by increasing cellular apoptosis and inducing caspase‐3 activity	Siddiqui et al. (2021)
Renal	Moringa seed oil	Interleukin‐1β (IL‐1β), tumor necrosis factor‐α (TNF‐α), and interleukin‐6 (IL‐6)	Decrease in serum creatinine and urea levels, decrease in oxidative stress by lowering of MDA, and elevation of renal SOD, CAT, and GPx activities	Edeogu et al. (2020)
Renal	Ethanolic extract moringa seed oil	Hepatic and renal markers in 2,2‐dichlorovinyl dimethyl phosphate	Increase in inflammatory markers, lowering of plasma protein, and alteration of plasma electrolyte	Adeoye et al. (2022)
Neuroblastoma	Moringa seed	SH‐SY5Y human neuroblastoma cells	Increase in the expression of p53, p21, and Bax, reduced nuclear translocation of NF‐κB	Cirmi et al. (2019)
Neuroblastoma	Moringa seed	SH‐SY5Y human neuroblastoma cells	Lower expression of NF‐kB, BACE1, APP and higher expressions of IkB and MAPT tau genes	Jaafaru et al. (2019)

Abbreviations: CAT, catalase; GPx, glutathione peroxidase; MDA, renal malondialdehyde; SOD, superoxide dismutase.

## 5 ANTIDIABETIC ROLE OF MORINGA SEED AND MORINGA SEED OIL

Diabetes mellitus (DM) is a metabolic disease that threatens life of world populations causing hyperglycemia that is the major cause of diabetic complications including retinopathy, neuropathy, and nephropathy. Moringa has been demonstrated in a variety of studies to have qualities that are beneficial for diabetics. According to the findings of an investigation, extracts of *M. oleifera* taken in water have been shown to be effective in treating both insulin‐resistant Type 2 diabetes and streptozocin (STZ)‐induced Type 1 diabetes in animals. Ground moringa seed brought about a reduction in the levels of glucose in the fasting blood of diabetic animals that had been induced by streptozotocin (STZ). In addition, when rats were given 500 mL of moringa seed powder for every kilogram (kg) of body weight (BW), there was a rise in the levels of antioxidant enzymes found in their blood. This suggests that the antioxidants found in moringa have the potential to suppress the generation of reactive oxygen species (ROS) in beta cells that have been activated by STZ. The production of superoxide and reactive oxygen species (ROS) in beta cells is a direct result of STZ‐induced adenosine triphosphate (ATP) dephosphorylation as well as the support provided by xanthine oxidase. Beta cells die out in those who have problems with their blood sugar levels being too high. When mitochondria receive an excessive amount of glucose, the production of reactive oxygen species is triggered. Apoptosis is triggered in beta cells because they have insufficient quantities of antioxidants. In the long run, this brings about hyperglycemia in addition to Type 2 diabetes as a result of a reduction in insulin production. Antioxidant flavonoids, such as quercetin and phenolics, are responsible for scavenging reactive oxygen species, also known as ROS. The flavonoids in moringa might remove mitochondrial ROS, protecting beta cells and bringing hyperglycemia under control (Gopalakrishnan et al., 2016).

In the latest investigation, 40 adult male Albino diabetic rats were given moringa seed flour, and its effects on them were analyzed. Two weeks were allotted to the process of familiarizing the rats to their restricted surroundings before the analysis began. They were split up into four groups: a control group, a group that received injections of STZ but no other therapy, and two treatment groups that received either a moderate dosage or a high dose of moringa seed powder. The control group served as the standard of comparison. The duration of the therapy was for a total of 4 weeks. At end of the research, the animals were put down and their organs, including their kidneys and pancreas, were taken for further analysis. Kidney homogenates were created so that researchers could evaluate factors, such as the extent of lipid peroxidation, the concentration of interleukin‐6 (IL‐6), and the performance of antioxidant enzymes. Additionally, blood was drawn for the purpose of biochemical analysis, which included the measurement of lipid peroxide, antioxidant enzyme activity, immunoglobulins (immunoglobulin G (IgG) and immunoglobulin (IgA)), IL‐6 concentration, glycosylated hemoglobin (HbA1c), liver enzymes, serum amylase, fasting blood sugar (FBS), serum albumin, renal functions (urea, creatinine, and urea nitrogen), serum electrolytes (sodium and potassium ions), and urine parameters. Physiological factors, such as food intake, water consumption, total body weight, food efficiency ratio, and organ weights, were measured and tracked during the experiment. Histological analysis was also performed on samples taken from the renal and pancreatic tissues. The diabetic rats were given crushed moringa seeds to evaluate what kind of reaction they had. According to the findings, giving rats moringa powder resulted in a significant decrease in the lipid peroxidation and an increase in levels of antioxidant enzymes in both the blood and the kidney tissue. The moringa therapy improved immunological function in addition to lowering levels of immunoglobulin and interleukin‐6 (IL‐6). In addition, supplementation with moringa led to a reduction in fasting blood sugar levels as well as glycosylated hemoglobin levels, both of which are indicative of better blood sugar management. After moringa was administered, there was a general improvement in blood electrolytes, as well as kidney function tests and urine analysis. In addition, supplementation with moringa led to improvements in organ weights, water consumption, overall body weight, and physiological markers. It was found that animals that were given ground moringa seed had a reduction in a variety of diabetes‐related symptoms and consequences (Ma et al., 2020).

In recent study, Alloxan monohydrate, 5,5‐Dithiobis (2‐nitrobenzoic acid) (DTNB), and a variety of other chemicals were used. Crude extracts were made by using methanol, the leaves and seeds of the *M. oleifera* plant were collected, dried, and then ground into a powder. In order to assess the presence and quantities of phenolic substances, flavonoids, alkaloids, and anthocyanidins, qualitative and quantitative phytochemical studies were performed on the extracts. The analysis was carried out using adult male Swiss albino rats. After the administration of Alloxan, which caused diabetes in the rats, several treatment groups were created. The levels of glucose in the mice’ blood were measured many times. Lipid peroxidation, NO, protein carbonyl (PC), reduced glutathione (GSH), and catalase (CAT) were the biomarkers that were tested in order to determine oxidative stress and antioxidant biomarkers. After the administration of extracts from *M. oleifera*, there was a considerable drop in the levels of glucose in the blood, with the biggest reduction happening when both the leaf and mehanolic seed extracts were applied at the same time. In addition, diabetic animals that were given the extracts did not see a reduction in their body weight. In response to the administration of the extracts, the size of the pancreas, kidneys, and liver all increased. It was found that there is a possibility that extracts of *M. oleifera* include antidiabetic capabilities. As a result, these extracts might reduce issues linked with diabetes (Aljazzaf et al., 2023).

In a similar experiment, when combined with feed, treatment with moringa seed powder at doses of 50 and 100 mg/kg BW for 4 weeks resulted in a 35% drop in fasting blood glucose and a 22% decrease in HBA1c compared to the control group. In STZ‐diabetic animals, the levels of IL‐6 and lipid peroxidation increased, but the activities of catalase, superoxide dismutase (SOD), and GSH decreased. On the other hand, diabetic animals/rats given with moringa in STZ showed virtually normal values for these parameters after treatment. Phytochemical analysis was not conducted for this investigation; nevertheless, polyphenols and flavonoids found in moringa seed powder have been shown to have antioxidant action (Al‐Malki & El Rabey, 2015).

In a recent investigation, the effects of *M. oleifera* on diabetes mellitus (DM) were revealed. There are a number of conditions that have been linked to variations in the INSR gene, including insulin resistance. The three‐dimensional (3D) structure of the insulin receptor (IR) protein has been uncovered. *M. oleifera* was selected because of its bioactive components and the advantages that it provides to health. After toxicity testing and interpretation of structures of phytochemicals, five compounds were selected for further study. These compounds were docked with the mutant protein by using the program PatchDock. Using pharmacophore models and LigandScout, the researchers were able to identify potential pharmacological targets that might be used in the treatment of diabetes. Both the structure and the interactions of a mutant insulin receptor kinase protein were studied. Mutations were found after researching the crystalline structure of the protein complex that was under investigation. After doing research, a selection of five phytochemicals derived from *M. oleifera* that interact with the protein was made. The results of the coupling were characterized by the presence of solid connections and good binding. A comparative study revealed that phytochemicals bind to the active site of the protein and lowered the risk of diabetes in body (Zainab et al., 2020).

Moringa seed oil plays an important role in the healing of wounds during diabetes. In a research, the effects of oleic acid and *M. oleifera* seed oil on the healing of lesions during diabetes were studied. The *M. oleifera* seed oil therapy has been shown to hasten the healing of wounds in mice in comparison to the control group; however, the oleic acid treatment hastens the healing of wounds even more. When compared to the vehicle group, the oleic acid treatment considerably decreased the size of wounds in immunocompromised rats. In diabetic rodents, the use of *M. oleifera* seed oil and oleic acid therapies hastened the healing of wounds. These therapies had very little impact on the levels of collagen and hydroxyproline. There is a lack of appropriate data about the usage of *M. oleifera* in the treatment of glycemic levels. A number of different processes play an important role, including the regulation of gene expression, the suppression of gluconeogenesis, the increase of insulin signaling, and the delay in stomach emptying. It will be necessary to conduct further intervention studies with uniform criteria, in addition to initiatives to quantify bioactive chemicals and explore extraction methodology. For therapeutic applications, it is necessary to have a full understanding of the composition of moringa extracts as well as the interactions between them (Nova et al., 2020).

## 6 ROLE OF MORINGA SEED AND MORINGA SEED OIL IN CARDIOVASCULAR DISEASE PREVENTION

Moringa seeds play an important role in the prevention of cardiovascular disease. The high antioxidant content of moringa seeds can help lower oxidative stress, which is a contributing factor in various illnesses including hypertension and heart failure. The presence of oleic acid in moringa seeds is one of the factors that contribute to their ability to lower the risk of cardiovascular disease as well as their impact of lowering blood pressure. This ability is one of the many notable benefits of moringa seed (Tadesse, 2019).

In a research, it was discovered that the bioactive compounds found in moringa seeds, including flavonoids, phenolic acids, and alkaloids, can lower the risk of developing cardiovascular illnesses because of their antioxidant and anti‐inflammatory qualities. Additionally, it has been established that the lipid‐lowering characteristics of moringa seeds can lessen the risk of atherosclerosis and other cardiovascular diseases. The use of moringa seeds can significantly lower the levels of cholesterol and triglycerides in the blood of rodents. Moringa seed has been shown to possess vasodilatory characteristics, which have been shown to enhance blood flow while simultaneously lowering blood pressure. It was found that hypertensive animals that were given an extract of moringa seed had considerably lower blood pressure (Oluranti, 2023).

In a similar in vivo (mice) experiment, after 28 days of oral administration of Moringa seed powder at doses of 600 and 900 mg, the values of left ventricular ejection fraction (LVEF) and left ventricular fractional shortening (LVFS) were raised in patients with contractile dysfunction. In mice with myocardial infarction (MI), moringa seeds prevented myocardial apoptosis, oxidative stress, and nitrosative stress, while also decreasing collagen deposition and boosting cardiac performance. Moringa seeds also improved cardiac function. It was revealed that moringa seeds have anti‐apoptotic and antioxidant capabilities, particularly in the context of myocardial infarction. Moringa seeds have been shown to provide anti‐apoptosis and antioxidant effects on heart function when given orally to mice that have been modeled after having a myocardial infarction. This impact is most likely the consequence of the seed's original chemical makeup, in addition to the additive effect of various antioxidant chemicals working together. Moringa seeds may offer therapeutic potential for minimizing myocardial infarction damage and other cardiovascular disorders. This potential comes from the seeds’ ability to reduce myocardial apoptosis, oxidative stress, and nitrosative stress (Li et al., 2020).

In another similar experiment, after a treatment period of 6 weeks, a methanolic seed extract of moringa at doses of 100 and 200 mg/kg BW dramatically lowered total cholesterol (TC) and very‐low‐density lipoprotein (VLDL) while simultaneously increasing high‐density cholesterol (HDL) cholesterol and preventing the cardiovascular diseases in mice. It was found that the low density lipopro (LDL) levels of the groups that were treated with moringa did not substantially differ from those of the control group (Ajayi et al., 2020).

In addition to this, the oil extracted from moringa seeds also provides protection against cardiovascular disease. According to the findings of a research project, the *M. oleifera* seed oil is mostly composed of oleic acid. The percentage of unsaturated fatty acids in the total amount of fatty acids was 79.86%, whereas the percentage of saturated fatty acids was 20.14%. Due to the high content of oleic acid in *M. oleifera* seed oil, the oil is extraordinarily stable and has a stronger oxidative stability than polyunsaturated fatty acids such as linoleic acid and linolenic acid. This is because oleic acid is monounsaturated, whereas linoleic acid and linolenic acid are polyunsaturated. Additionally, oleic acid may have beneficial effects on nutrition and may make a substantial contribution to the prevention of cardiovascular disease (Fu et al., 2021).

In a different study, rats’ absolute heart weight and myocardial histoarchitecture were highly enhanced by *M. oleifera* seed oil. Absolute cardiac weight and the histology of the cardiac muscle were partially recovered after the injection of *M. oleifera* seed oil. On the other hand, *M. oleifera* seed oil considerably reduced MDA formation while also increasing cardiac SOD and glutathione peroxidase (GPx) activity. The antioxidant properties of *M. oleifera* seed oil were responsible for this. This nutritional molecule's cardioprotective action may be due to its antioxidant capability and can be attributed to its phytochemical components, especially its high flavonoid content. Flavonoids are known to alter redox status by boosting the endogenous antioxidant defense capacity, particularly SOD, catalase, and GPx. Superoxide radical anion is dismutated by SOD, whereas catalase and glutathione peroxidase (GPx) convert hydrogen peroxide (H_2_O_2_) to H_2_O (Saka et al., 2020).

## 7 ANTI‐OBESITY ROLE OF MORINGA SEED AND MORINGA SEED OIL

Obesity can cause an oxidative stress situation because pro‐oxidants and antioxidants of the body are out of balance. There is increased lipogenesis and lipolysis inhibition in obesity. There are more glucocorticoid and androgen receptors, a higher metabolic rate, sensitivity to lipolysis, and a higher risk of insulin resistance in fat, particularly in the visceral area during obesity. This circumstance has the potential to result in dyslipidemia, insulin resistance, and inflammation that compromise endothelial function (Madkhali et al., 2019).

Moringa seeds contain a number of bioactive chemicals that have antiobesity characteristics. These compounds, which include flavonoids, phenolic acids, and alkaloids, can help people lose weight and prevent issues associated with obesity. Additionally, it has been revealed that moringa seeds contain lipid‐lowering effects, which can assist in the prevention of fat formation and obesity. A research found that administering an extract made from moringa seed resulted in significant reductions in the body weight, body mass index (BMI), and percentage of body fat in obese rats (Ezzat et al., 2020).

In the latest investigation, five groups of five rats each weighing 120–150 g were fed a high fat diet (HFD) for 10 weeks in order to become obese. After establishing the baseline lipid profile, treatment with methanol extracts of *M. oleifera* seed extract (MOSE) at 100 and 200 mg/kg BW for 6 weeks started. The Orlistat‐treated (50 mg/kg BW), untreated, and regular diet groups served as the control groups. In vivo antihyperlipidemic activity and an enzyme antilipase assay were determined. In comparison to the regular Orlistat, there was a significant reduction in very‐low‐density lipoprotein cholesterol (VLDLc) across the five groups (*p* < .01). In the group receiving 200 mg/kg MOSE, a statistically significant rise in high‐density lipoprotein cholesterol (HDLc) was seen (*p* < .01). However, in the group receiving 100 mg/kg MOSE, a gravely significant rise in the atherogenic index (AI) was seen. The methanolic extract of *M. oleifera* seed was capable of changing lipid profiles (Sivanesan et al., 2022).

Moringa seed oil also reduces obesity risk. In an experiment, 48 male rats were divided equally into six groups. Group Ι (C) served as control, group ΙΙ (MC) was given *M. oleifera* seed oil extract (800 mg/kg BW) for 8 weeks, group ΙΙΙ (LC) was given (20 mg/kg BW) LYC for 8 weeks, group ΙV (O) received high fat diet (HFD) for 20 weeks, group Ѵ (MO) was given HFD for 20 weeks and received (800 mg/kg BW) *M. oleifera* seed oil extract for last 8 weeks, and group ѴΙ (LO) received HFD for 20 weeks and was given (20 mg/kg BW) LYC for last 8 weeks. In HFD‐fed rats, the extract from the seeds of *M. olifera* decreased the levels of non‐esterified fatty acid (NEFA), dyslipidemia, and hyperglycemia. The phytochemicals found in *M. olifera*, including phenolic, flavonoids, and saponins, may be responsible for the plant extract's antidyslipidemic and hypoglycemic benefits against obesity. These compounds are crucial for regulating lipid levels (Kilany et al., 2020). Table 2 presents the health benefits of moringa seed and moringa seed oil in the light of recent studies.

**TABLE 2:** Health benefits of moringa seed and moringa seed oil in the light of recent studies.

Type of disease	Source/intervention	Study type/model	Effect/result	References
Inflammation	Moringa seed oil	Inflammatory cytokine TNF‐a	Sterol compound acts as an anti‐inflammatory agent by obstructing pro‐inflammatory cytokine TNF‐α	Famurewa, Aja, et al. (2019); Famurewa, Asogwa, et al. (2019)
	Moringa seed oil	Pro‐inflammatory cytokines, such as IL‐1α, IL‐12P70, IL‐17A, IL‐6, IL‐1β, and TNF‐α and anti‐inflammatory cytokines (IL‐4, IL‐13, and IL‐10)	Increase in anti‐inflammatory properties because of the presence of phytochemicals and unsaturated fatty acids	Shamlan et al. (2021)
	Moringa seed extract	iNOS, IL‐1β, and IL‐6 cell lines	Lower carrageenan‐induced rat paw edema (33% at 500 mg/kg MIC‐1) comparable to aspirin (27% at 300 mg/kg)	Adji et al. (2022)
	Ethanolic extracts of young leaves, seeds, and roots of moringa	Murine macrophage RAW264.7	The positive correlation between flavonoid content and anti‐inflammatory and antioxidant activities	Ramamurthy et al. (2021)
Diabetes	Moringa seed powder	Rats were intravenously injected with streptozotocin (60 mg/kg body weight)	Low doses of moringa contain antidiabetic activity due to its content glucomoringin, phenols, and flavonoids	Ma et al. (2020)
	Moringa leaf and seed extract	Rats were injected with alloxan	Reduction in fasting blood glucose to normal levels, decrease in cholesterol, triglycerides, creatinine, and liver enzymes	Aljazzaf et al. (2023)
	Moringa seed extract	Rats were injected with streptozotocin with 50 and 100 mg/kg	Decreased FPG, and increased SOD, CAT, and GSH in serum and kidney with positive effect on diabetes	Waterman et al. (2020)
Oxidative stress	Moringa seeds	Oral administration of benzene in Wistar rats	LPO and antioxidant markers [SOD, TRG, GPx, and CAT] come to normal value	Rajkumar et al. (2022)
	Moringa seeds	Selenium (Se)‐enriched antioxidant peptides, that is, FLSeML, LSeMAAL, LASeMMVL, SeMLLAA, and LSeMAL	Higher cell viability, reduced reactive oxygen species accumulation, increased superoxide dismutase and catalase activities	Chen et al. (2023)
	Moringa seed extract	Wound healing potential of Moringa seed extract was investigated in male albino rabbits	Higher wound healing rate, elevating TGF‐β1, VEGF, Type I collagen relative expression, and reducing inflammatory markers, such as IL‐1β and TNF‐α	Shady et al. (2022)
Cardiovascular disease	Moringa seed	Mice fed with foods containing Moringa seed powder	Inhibition of MI‐induced apoptosis, reduction of cardiac fibrosis, suppression of oxidative and nitrosative stress	Li et al. (2020)
	Moringa seed oil	Mice fed with foods containing Moringa seed oil	Significant decreases in body weight gain and cardiac weight in DDVP‐exposed animals (*p* < .05) with increase in malondialdehyde, reduction in superoxide dismutase and glutathione peroxidase	Saka et al. (2020)
Obesity	Methanolic extract of moringa seed	Methanol extracts of Moringa seed at 100 and 200 mg/kg b w for 6 weeks	Significant decrease in VLDLc at *p* < .01, significant increase in HDLc at *p* < .01	Ajayi et al. (2020)
	Moringa seed oil	Moringa seed oil at a dosage of 0.5 mL/kg for 8 weeks in rats	Significant reduction in TC, LDL and increased HDL levels in obese rats	Sivanesan et al. (2022)
	Moringa seed oil	Moringa seed oil at a dosage of 800 mg/kg b.wt for 20 weeks	Administration of the moringa seed oil extract and LYC has antiobesity potential in HFD‐induced obesity in rats	Kilany et al. (2020)

Abbreviations: CAT, catalase; GPx, glutathione peroxidase; HDLc, high‐density lipoprotein cholesterol; HFD, high fat diet; LPO, lipid peroxidation; LYC, lycopene; SOD, Superoxide dismutase; TRG, total reduced glutathione; VLDLc, very‐low‐density lipoprotein cholesterol.

## 8 CONCLUSION

In conclusion, this review highlights the inherent health benefits associated with the consumption of moringa seeds. The fatty acid found in the highest concentration in moringa seed oil is oleic acid (71.57%). followed by palmitic acid (8.22%), then stearic acid (5.25%), and behenic acid (4.15%). The compositional characteristics of moringa seed oil, characterized by its notable abundance of oleic acid, provide a desirable lipid profile, thereby implying potential advantages for cardiovascular health. The distinct activation of caspases was observed upon the presence of moringin and avenanthramide, thereby resulting in a notable decrease of cancer cell proliferation. The utilization of moringa seeds has been scientifically demonstrated to provide advantageous physiological effects including capacity to prevent ROS generation associated with diabetes (hyperglycemia) and lower cholesterol levels. The methanol/ethanolic extracts of *M. oleifera* seed are capable of changing lipid profiles. The phytochemicals found in *M. oleifera*, including phenolic, flavonoids, and saponins, may be responsible for the plant extract's antidyslipidemic and hypoglycemic benefits against obesity and cardiovascular diseases. This exemplifies the remarkable versatility of moringa as a plant with medicinal properties, as it exhibits the capacity to confer advantageous effects across numerous health‐related contexts. It is possible that moringa seeds could emerge as a highly advantageous addition within the list of naturally existing compounds that exhibit promising prospects for utilization in medicinal contexts. It is vital to undertake further investigations to validate these findings and explore the in vivo impact of moringa seeds, alongside clinical trials including human subjects.

## AUTHOR CONTRIBUTIONS

**Muhammad Shahbaz:** Writing – original draft (equal). **Hammad Naeem:** Writing – original draft (equal). **Maryam Batool:** Writing – review and editing (equal). **Muhammad Imran:** Conceptualization (equal); resources (equal). **Muzzamal Hussain:** Writing – review and editing (equal). **Ahmad Mujtaba:** Data curation (equal). **Suliman A. Alsagaby:** Investigation (equal); methodology (equal). **Waleed Al Abdulmonem:** Data curation (equal); software (equal). **Ahmed H. El‐Ghorab:** Conceptualization (equal); validation (equal); visualization (equal). **Mohammed M. Ghoneim:** Resources (equal); writing – review and editing (equal). **Mohamed E. Shaker:** Data curation (equal); project administration (equal). **Mohamed A. Abdelgawad:** Writing – review and editing (equal). **Entessar Al Jbawi:** Data curation (equal); supervision (equal).

## CONFLICT OF INTEREST STATEMENT

The authors declare no conflict of interest.

## Data Availability

The data that support the findings of this study are available from the corresponding author upon reasonable request.
